# Transition readiness in adolescents and young adults with chronic rheumatic disease in Oman: today’s needs and future challenges

**DOI:** 10.1186/s12969-022-00687-6

**Published:** 2022-04-12

**Authors:** Reem Abdwani, Rumaitha Al Sabri, Zawan Al Hasni, Seyad Rizvi, Humaid Al Wahshi, Batool Al Lawati, Safiya Al Abrawi, Yassir Wali, Mona Al Sadoon

**Affiliations:** 1 Department of Child Health, College of Medicine & Health Sciences, Sultan Qaboos University, P.O. Box 35, Al Khoudh 123, Muscat, Oman; 2 Pediatric Residency Program, Oman Medical Specialty Board, Muscat, Oman; 3 General Foundation Program, Oman Medical Specialty Board, Muscat, Oman; 4 Department of Public Health, College of Medicine and Health Sciences, Sultan Qaboos University, Muscat, Oman; 5 Department of Medicine, Royal Hospital Muscat, Muscat, Oman; 6 Department of Medicine, College of Medicine and Health Sciences, Sultan Qaboos University, Muscat, Oman; 7 Department of Child Health, Royal Hospital Muscat, Muscat, Oman

**Keywords:** Transitional care, Adolescents, Oman

## Abstract

**Introduction:**

In Oman, the ““transition” of health care of adolescents to adult care occurs at a young age, like many other GCC countries for cultural reasons. In order to address this concern, this study was conducted to determine the transition readiness skills of adolescents and young adults with childhood onset rheumatic diseases using a cross-cultural adaptation of the UNC TRxANSITION scale.

**Methods:**

We used a professionally translated/back translated, provider-administered UNC TR_x_ANSITION Scale. This 32-question scale measures HCT in 10 domains including knowledge about diagnosis or treatment, diet, reproductive health, school/work, insurance, ability to self-manage and identification of new health providers. The maximum transitional score of 10, was categorized as low (1-4), moderate (4 - 7) and high (7 -10) transitional readiness scores.

**Results:**

We enrolled 81 Omani adolescents and young adults (AYA) with chronic childhood onset rheumatic diseases. The cohort consisted of 79% females, with mean age of 15.8 years (± 3.53) and mean disease duration of 6.95 years (± 4.83). Our cohort’s overall mean score is low 5.22 (±1.68). Only 14.8% of the cohort achieved a high transition score (≥7). Significant direct relationship was observed between age and the mean transition readiness score (*r* = .533, *P* < .001). The mean transition readiness score in the younger age group (10-13 years) was 4.07 (±1.29), the middle age group (14-18 years) was 5.43 (±1.27), while the older age group (19-21 year), was 6.12 (±1.81). Mean transition score of youngest age group was found to be significantly lower than the other two age groups (*p* = .003).

**Conclusion:**

Overall, the transition readiness of AYA in Oman is low compared to other western countries indicating the need to initiate a health care transition preparation program for patients with chronic diseases across the country. In addition, we need to establish regional guidelines to address the transfer and transition policies to be in line to international recommendations.

As transition continues after transfer, and is preferably guided by adolescent developmental status rather than chronological age, it would be preferable to refer to the transition and transfer policies 9rather than transitional age policy) to be in line to international recommendations.

## Introduction

Advances in health care over the last decades have led to substantial improvement in the outcome of young people with childhood onset rheumatic diseases due to the availability of more efficacious therapies and improved treatment strategies [1–5]. However, the transition of adolescents and young adults (AYA) with rheumatic diseases continues to be challenging and complex. Over half of the young people transferred to an adult rheumatologist have inadequate follow-up [6]. This is worrisome, especially that over 50% of young people with JIA enter adult care with flare of disease, whereas a significant proportion of young people with childhood onset SLE develop disease flare within 1 year of transitioning to adult care [7–9]. Also, young adults with rheumatic disease are less likely to have college education, maintain employment and tend to have lower income than their peers [10, 11]. Hence, effective transitional care for AYA with rheumatic disease is fundamental to rheumatology care provision.

Health care transition (HCT) is defined as the purposeful and planned movement of adolescents and young adults with chronic physical and medical conditions from child-centered to adult oriented healthcare systems [12]. Transition of care is a multifaceted active process that focuses on the medical, psychosocial, educational, and vocational needs of adolescents as they move from child to adult centered care. This is in contrast to transfer of care, which is an event that occurs at a specified point in time rather than a process. Transition programs for adolescents with chronic diseases aim to provide comprehensive, coordinated, uninterrupted health care that is age and developmentally appropriate. They promote skills in communication, decision making, and self-care and therefore enhance a young person’s control and independence. HCT can be characterized by a three stage process 1) preparation phase, starts in early adolescence, 2) transfer of care phase, usually in late adolescence and 3) integration into adult health care phase, which continues following the transfer [13]. A fundamental element of the HCT process is preparing young adolescents how to manage their own health. This involves the adolescent, family members, as well as the pediatric and adult healthcare provider as equally engaged collaborators. By using transition readiness assessment tools, providers can objectively evaluate the knowledge and skills required for adolescent and young adults to manage their own health.

There are several validated transition readiness assessment tools for AYA with chronic diseases.

have been described in the literature. A recent systematic review identified 19 tools in the literature, including the Transition Readiness Assessment Questionnaire [14, 15], the UNC TRxANSITION Scale [16], the STARx Questionnaire [17], the Am I ONTRAC for Adult Care questionnaire [18], TRANSITIONQ [19], and the Adolescent Assessment of Preparation for Transition [20]. Among the 19 tools, the UNC TRxANSITION Scale, is the only tool that is administered to the adolescent by a healthcare provider; the other 18 tools are self-reported [21]. The UNC TRxANSITION Scale is a disease-neutral tool that can be used in the clinical setting. It is a reliable and valid tool that measures health-care transition knowledge, skill mastery and monitors progression in multidimensional fashion [22]. A recent systematic review on transition readiness showed that UNC TRxANSITION Index scale has been used by several studies [23, 24].

Within the healthcare system in Oman, like many other GCC countries in the region, transitional health care is not well developed. There is no standard transition process for young people with chronic diseases. The “transfer” of care adolescents to adult care is an “event” that occurs at the age 12-13 years, like many other Arabic/Muslim countries for cultural reasons. There is also paucity of literature on transitional care of young people with chronic disease from this region. In order to address this issue, the aim of this study is to determine the transition readiness skills of young people with childhood onset rheumatic diseases in Oman using a cross-cultural adaptation of the UNC TRxANSITION Scale.

## Method

### Instrument and validation

The UNC TRxANSITION Scale™ is validated and reliable in both the pediatric and adult patients. The scale is composed of 32 questions which are distributed in 10 domains which are knowledge on 1) Type of illness *(T),* 2) Medications *(Rx)*, 3) Adherence *(A),* 4) Nutrition *(N),* 5) Self-management skills *(S),* 6) Informed reproduction issues *(I),* 7) Trade/school issues *(T),* 8) Insurance issues *(I),* 9) Ongoing support *(O)* and 10) New health care provider identification *(N).* In this scale, the total score of transition readiness and domain scores are computed based on clinician ratings of patient responses. Each question in the scale is scored as follows: 0 points indicates no knowledge or self-management skills, 0.5 points means some knowledge or self-management skills, or 1 point reveals complete knowledge or self-management skill. A total score is calculated ranged between 0 and 10, while the transition readiness assessment scores were categorized as low (0 - 4), moderate (4 - 7) and high (7 -10). For the study, trained research assistants administered the questions of UNC TRxANSITION Scale in the form of an interview. We ensured that the AYA answered these questions independently without the input of their parents by interviewing them in a private room.

The original English-language version of the UNC TRxANSITION Scale was translated to Arabic language, for better communication with patients, using established forwards and backwards translation methods. Two independent translators each translated the English language version of the scale into Arabic. Then a committee of Arabic-speaking researchers compared these translations both with each other and with the original English version in order to create a first draft. Next, the two independent translators translated the draft back into English and the committee again compared both back-translated versions together and to the first draft to ensure the accuracy of the content. A second draft was then created and examined to resolve any ambiguities. The second draft, along with the original English-language version of the UNC TRxANSITION Scale, was submitted to three pediatricians and two physicians to check face validity. Some minor modifications were made based on their feedback. The final Arabic version of the scale was deemed to reflect an accurate translation of the items in the original UNC TRxANSITION Scale. A pretesting of the instrument was subsequently conducted among 10 healthy children (six males and four females) to assess the clarity of the items in the final Arabic version of the scale and estimate the time required for the interview. The results indicated that the Arabic scale was well understood by all and was time efficient, taking approximately 20 min to administer.

### Data collection

After obtaining informed consent, we recruited (*n* = 81) Omani AYA from Sultan Qaboos University Hospital and Royal Hospital, between 10 to 21 years of age and diagnosed with a chronic rheumatic condition for at least 1 year. We recruited a similar number of young people in three age groups; younger (10 -13 years), middle (14-18 years) and older (19-21 years) age groups. The diagnoses of our cohort included juvenile idiopathic arthritis, systemic lupus erythematosus and other childhood onset rheumatic diseases such systemic vasculitis and juvenile dermatomyositis. Exclusion criteria were major cognitive disorders or any comorbidity that interferes with future self-management of health. The information collected on sociodemographic factors included gender, age, type of school, mother’s education level, father’s education level, mother’s occupation, father’s occupation, type of family, order among siblings, presence of the disease in siblings and income was collected.

### Statistical analysis

Data base for the study was created in IBM SPSS 23 software. In the UNC TRxANSITION Scale for AYA; as all questions were not applicable for all subjects, hence, the proportion score for the subjects was obtained by including only the relevant questions for the respective subjects. Kolmogorov-Smirnov test was used to test the normality of the variable in different categories of the demographic characteristics. Independent sample t-test or ANOVA were used to test the significance of the difference between the observed means of the categories, if the distribution pattern was found normal, otherwise Mann-Whitney U or Kruskal Wallis H tests were applied, respectively. Pearson Correlation formula was used to evaluate the degree of linear relationship between age and transition readiness score. *P* ≤ .05 has been considered as significant.

## Results

The study sample (*n* = 81), was distributed into three age groups of similar numbers; (10-13 years (*n* = 26), 14-18 years (*n* = 29) and 19-21 years (*n* = 26). The mean age of the cohort was 15.80 years (± 3.53), the mean age of disease onset was 8.81 years (± 5.51) with mean disease duration of 6.95 years (± 4.8 years). A total of 64 participants were female (79%). Majority of patient originate from Muscat region (*n* = 27, 33%), followed by Al Shariqiya and Al Batina region (*n* = 21, 26%) equally. The cohort consist of young people diagnosed with childhood onset systemic lupus erythematosus (*n* = 50, 62%), juvenile idiopathic arthritis (*n* = 23, 28%), and other chronic childhood onset rheumatic diseases (*n* = 8, 10%). There was no significant difference between age groups in all sociodemographic variables; hence the results are described for the whole sample in Table 1. Father’s educational level was college degree or higher in 41%, while 68% were employed. The mother’s educational level was college degree or higher in 28%, while 21% were employed. The total household monthly income was less than 1000 Omani Rials ($2500 USD) in 53% of the cohort. In general, the demographics of our cohort was overall representative of the population demographics. Although, the household monthly income is somewhat lower than reported average monthly income, 1550 RO ($4000 USD) in Oman.

**Table 1:** Sociodemographic of adolescent and young adults with chronic rheumatic diseases

	Frequency (*n* = 81)	percentage
Gender		
Male	17	21%
Female	64	79%
Age groups		
10-13 years	26	32%
14-18 years	29	36%
19-21 years	26	32%
Region		
Muscat	27	33%
Al Sharqiya	21	26%
Al Batina	21	26%
Al Dakhlia	5	6%
Muscandam & Al Wusta	4	5%
Dhofar	3	4%
Father Educational Level Illiterate		
School	14	17%
College and higher	34	42%
Employment	33	41%
	55	68%
Mother Education Level		
Illiterate	17	21%
School	41	51%
College and higher	23	28%
Employment	17	21%
Household income/month		
< 1000 RO	43	53%
1000 -2000 RO	29	36%
> 2000 RO	9	11%
Transition readiness score		
Low score (0-4)	18	22%
Medium score (4-7)	51	63%
High score (7-10)	12	15%

**Fig. 1:**
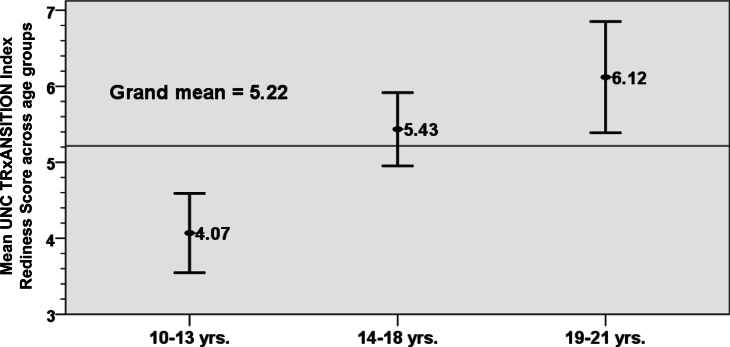
Mean and 95% confidence interval of Transition Readiness Index Score in different age

There was a statistical significance within the transitional readiness index score across age groups as shown in Fig. 1. The overall mean score for the sample is 5.22 (±1.68). The mean scores of our cohort belonging to younger, middle and older age groups were all in the ‘moderate category’ of transition readiness scale [4–7]. However, there is a steady increase in the overall mean score with increase in age groups, with the mean score of 4.07 (±1.3) in the younger, 5.43 (±1.27) in the middle and 6.12 (± 1.8) in the older age group (*p* < 0.001). Further analysis of the three age groups as displayed in Table 2. The overall cohort (*n* = 81) achieved a transition readiness index score was defined as low in 22%, medium in 64% and high in 14.8%. (*p*_=_ 0.0004). The cohort with a high transition score, majority belonged to the older group while only one of the patients in the younger age group had a high transitional index score. Interestingly, in the older age group, a high transition readiness score was achieved in only 30.8% of the cohort, while the majority had a moderate score 61.5%.

**Table 2:** Transitional readiness index score categories in different age groups

Transitional readiness categories	Age group (*n* = 81)			Total
	10-13 yrs	14-18 yrs	19-21 yrs	
Low score (0-4)	(*n* = 12) 46.2%	(*n* = 4) 13.8%	(*n* = 2) 7.7%	(*n* = 18) 22.2%
Medium score (4-7)	(*n* = 13) 50.0%	(*n* = 22) 75.9%	(*n* = 16) 61.5%	(*n* = 52) 64.2%
High score (7-10)	(*n* = 1) 3.8%	(*n* = 2) 9.5%	(*n* = 9) 26.5%	(*n* = 12) 14.8%
Total	26	29	26	81

**Fig. 2:**
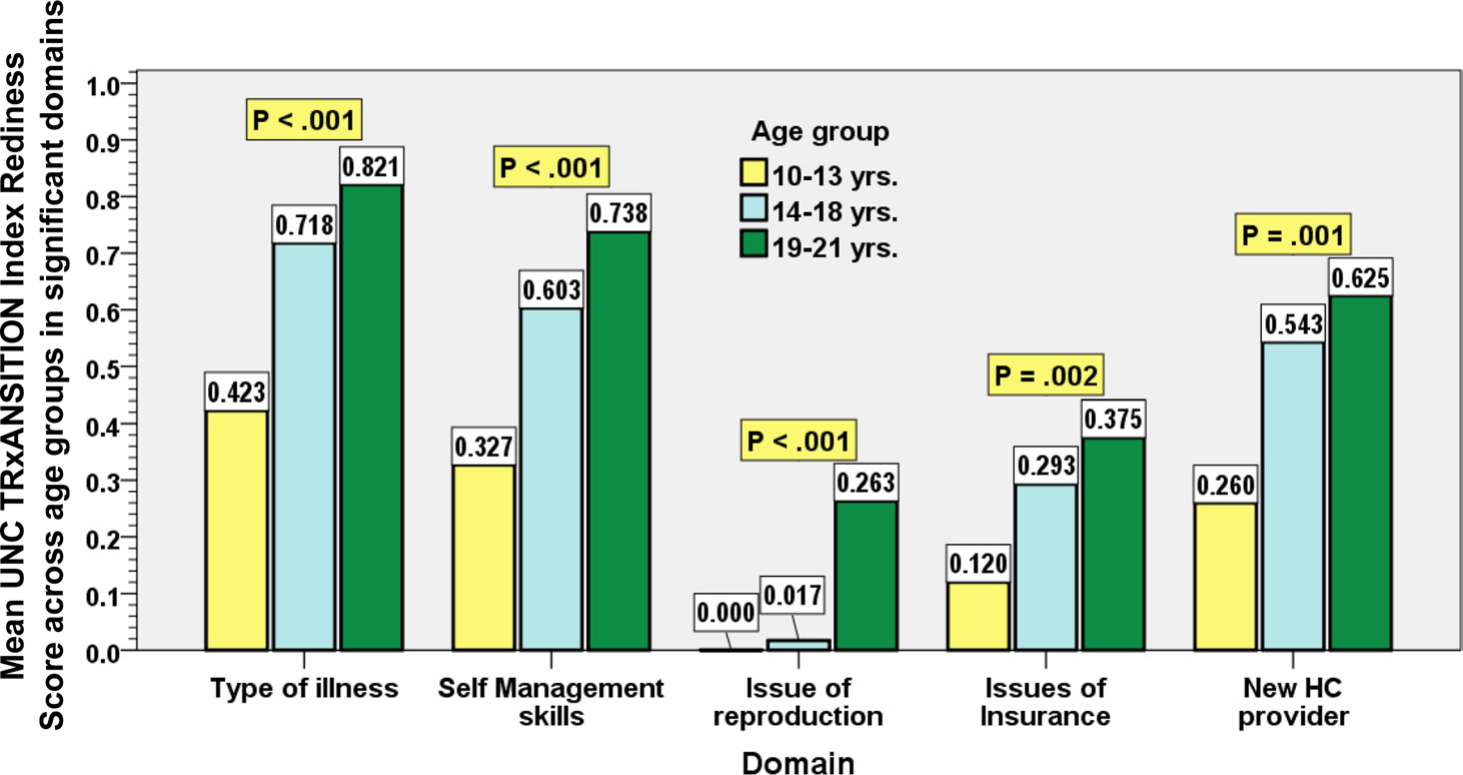
Mean TIS in significant domains in different age groups

**Fig. 3:**
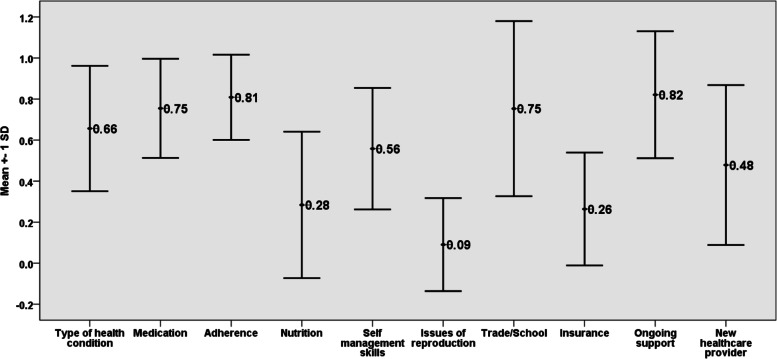
Mean UNC TRxANSITION Scale in 10 subdomains

The detailed comparison of the statistically significant mean transitional scores across different age groups in the subdomains of UNC TRxANSITION Scale domains are highlighted in Fig. 2. For the overall mean transition score, a statistically significant difference was found between the age groups (*p* < 0.0004) and the difference in mean scores between any two age groups on post-hoc pairwise analysis was also statistically significant. The post-hoc pairwise analysis locates the differences in scores between any two age groups across the 10 domains are displayed in Table 3. In section wise analysis of the 10 subdomains across the three age groups, the statistically significant sections include: Type of illness, Self-management skills, Issues of reproduction, Issues of insurance and new healthcare provider identification as displayed in Table 3 and Fig. 2. As Fig. 3 depicts, the transition index score was the highest in the following domains; Adherence 0.81 (SD 0.21), Medication 0.76 (0.24) and Trade/School 0.75 (0.43); while the following domains had the lowest transition readiness index score: Issue on reproduction 0.09 (SD 0.22), Insurance 0.26 (SD 0.28) and Nutrition 0.28 (0.36).

**Table 3:** Mean UNC TRANSITION readiness index and Post Hoc comparison among different age groups

Characteristic	Age groups	Mean ± SD	***p*** value	***p***- values for Post-hoc comparisons
*Type of illness*	a	0.42 SD (0.25)	***P*** **= 0.000**	a & b (*p* = 0.002)
	b	0.69 SD (0.24)		a & c (*p* = 0.000)
	c	0.81 SD (0.27)		b & c (*p* = 0.203)
*Medications*	a	0.69 SD (0.24)	***P*** **= 0.245**	a & b (*p* = 0.587)
	b	0.76 SD (0.246)		a & c (*p* = 0.217)
	c	0.789 SD (0.23)		b & c (*p* = 0.853)
*Adherence*	a	0.78 SD (0.23)	***P*** **= 0.698**	a & b (*p* = 0.896)
	b	0.81 SD (0.177)		a & c (*p* = 0.673)
	c	0.828 SD (0.207)		b & c (*p* = 0.943)
*Nutritional restrictions*	a	0.288 SD (0.325)	***P*** **= 0.845**	a & b (*p* = 0.959)
				a & c (*p* = 0.950)
	b	0.317 SD (0.387)		b & c (*p* = 0.833)
	c	0.26 SD (0.369)		
*Self-management skills*	a	0.327 SD (0.247)	***P*** **= 0.000**	a & b (*p* = 0.003)
	b	0.567 SD (0.27)		a & c (*p* = 0.000)
	c	0.729 SD (0.22)		b & c (*p* = 0.048)
*Issues of Reproduction*	a	0.00 SD (0.00)	***P*** **= 0.000**	a & b (*p* = 1.000)
	b	0.00 SD (0.00)		a & c (*p* = 0.000)
	c	0.216 SD (0.31)		b & c (*p* = 0.001)
*Trade/ School issues*	a	0.788 SD (0.40)	***P*** **= 0.563**	a & b (*p* = 0.599)
	b	0.667 SD (0.48)		a & c (*p* = 0.996)
	c	0.779 SD (0.41)		b & c (*p* = 0.612)
*Insurance issues*	a	0.12 SD (0.17)	***P*** **= 0.002**	a & b (*p* = 0.094)
	b	0.28 SD (0.255)		a & c (*p* = 0.001)
	c	0.36 SD (0.308)		b & c (*p* = 0.470)
*Ongoing support*	a	0.769 SD (0.29)	***P*** **= 0.577**	a & b (*p* = 0.602)
	b	0.857 SD (0.32)		a & c (*p* = 0.672)
	c	0.838 SD (0.319)		b & c (*p* = 0.974)
*New healthcare provider identification*	a	0.260 SD (0.295)	***P*** **= 0.002**	a & b (*p* = 0.009)
	b	0.583 SD (0.338)		a & c (*p* = 0.003)
	c	0.581 SD (0.42)		b & c (*p* = 1.00)

Group Identity: a is 10-13 years (lower); b is 14-18 years (middle) and c is 19-21 years (older)

## Discussion

The unmet training needs of healthcare professional in adolescent medicine is a global as well as a regional challenge [25–27]. Adolescents make up more than 25% of the population of Gulf Cooperation Council (GCC) countries, a percentage that is far higher than that in other high-income countries, yet their health status and health care needs are not given proportional attention in the region [28]. While there is progress in the health systems in GCC, a gap exists between the training, knowledge and skill-set of existing health-care providers and the needs of the adolescents for whom they provide care [29, 30]. Many health care providers in the region have limited or no training in adolescent health care. Therefore, adolescents tend to be “transferred” rather than “transitioned” to the adult care system at a very young age (12-13 years) due to cultural reasons without the necessary health care transition process. This poses a challenge on the outcome of adolescents with chronic disease in the region. In fact, a recent systematic review suggests that mandating or recommending a delayed age of transfer constitutes a simple but effective means of improving transition for young people with chronic conditions across all clinical condition [31].

There are major differences in the pediatric and adult health care models that need to be understood to manage the expectations of both patients and healthcare providers for the transitional process to be successful. Pediatric care tends to be a family-centered approach, whereas adult care tends to be patient centered. Similarly, pediatric care is developmentally focused and adapts to the growing needs of children and adolescents, whereas adult care tends to be more disease focused. Moreover, pediatric team approach tends to be multidisciplinary, whereas adult care is reliant on referrals to other services. In addition, the clinical settings are perceived as nurturing, and they focus on psychosocial support for adolescents and their families, whereas adult services offer a more cognitive and information-based approach to care [32, 33]. These differences in approaches to care require adjustment and skills in self-care and advocacy that adolescents must develop to transition successfully from the pediatric health care system to the adult health care system. However, since the “transfer of care” in Oman, like many other GCC countries, occurs at an early age, this adjustment process does not develop for both AYA as well as healthcare providers.

Based on the joint clinical recommendations from the American Academy of Pediatrics (AAP), the American Academy of Family Physicians (AAFP), and the American College of Physicians (ACP), Got Transition/Center for Health Care Transition Improvement developed an evidence-informed model, Six Core Elements of Health Care Transitions [34]. It consists of the following steps 1) discussing the transition guidelines (12-14 years), 2) transition tracking and monitoring (14-18 years), 3) transition readiness assessment with validated tools (14-18 years), 4) transition planning (14-18 years), 5) transfer of care (18-21 years), and finally tracking 6) transfer completion (18-23 years). In our cohort AYA who are followed at the two main pediatric and adult rheumatology centers in Oman, who have had no formal transitional services, the transfer event occurs at the age of 13 years, which is the suggested age to begin the discussion of transitional policy and process in most western countries rather than the actual transfer that occurs in this region of the world.

Similar to other pediatric rheumatology studies, transitional readiness score increases with advancing age [35, 36]. In fact, transitional readiness skills continue to develop post transfer into adult care [37]. In our study, the overall mean transitional score for our cohort was 5.22 (SD 1.68) which is overall lower than other studies using UNC TR_x_ANSITION Scale transitional tool assessment [22–24]. In our cohort, the mean transitional score at the current ‘transfer’ age is 4.07 (± 1.27) which is considered to be a low score, only one person in this age group had a high transitional score. This young person had a disease onset at 3 years with disease duration over 10 years at the time of transfer, in addition to both parents having a high educational level, which might have contributed to the favorable results. However, there is a gradual trend in increase in the mean transitional score across age groups with a transitional score of 6.12 (± 1.81) in the older age group. Despite the increasing trend of transitional scores, only 30.8% of patients in the older group were considered to have a high transitional score. Of the 10 subdomains in UNC TRxANSITION scale with lowest score included the domains of Issues of Insurance and Issue of Reproduction. Given that healthcare service is free to all Omani citizens within government institutions, the relevance of insurance is applicable to Omani citizens seeking services in private hospitals. Some companies offer insurance to employees while others extend the insurance to cover family members. This might have caused confusion in the insurance domain as AYA were not aware of this issue. With regards to Issue of Reproductions, this is a sensitive matter that most AYA in the region do not feel at ease to discuss. In the Middle East, school based sexual educational programmes are not implemented due to cultural barriers and considered the responsibility of the parents. Hence, given the cultural sensitivity with regards to discussing sexual and reproductive health with adolescents, the Issues of Reproduction, were addressed to females over 18 year of age or who were married. Despite this, the knowledge of the older group in this domain appears to be the lowest of all other subdomains.

Overall, our results reflect that our AYA patients with chronic rheumatic diseases are not adequately prepared for the ‘transfer’ to adult care at the current cut-off age of 13 years. It emphasizes the need to initiate transitional services within the country, which should commence by increasing the transfer age to 18-21 years as evidenced by the results of our study and keeping in par with international recommendations [38]. Similarly, the relatively low percentage of young people in the older group with a high transitional score, suggest that further educational measures at an earlier age are needed to improve the transitional process. Greater emphasis in education should be in domains with the lowest mean transitional scores. The implementation of transitional care into practice in Oman needs to be evidence based and identifies effective measures of transitional care and quality indicators to measure success.

The lack of transition care services is a global challenge and not limited to this region of the world. In a recent survey that focuses on current practices in transitional care among 115 pediatric rheumatology centers in 22 European Union countries, 23% responded that their centers did not offer transition services, however, the majority agreed that a formal process in transitioning patients to adult care is necessary [39]. Similarly, a recent survey in North America among pediatric and adult rheumatologists was conducted among Childhood Arthritis and Rheumatology Research Alliance (CARRA) members. Of the 217/398 members responded, 63% did not consistently address healthcare transition with patients, only 17% had a transition policy and only 31% used a transitional tool, while a dedicated transition clinic was available in 23% of centers. In contrast to our study, the most common age to begin transition planning was 15-17 (49%), and most providers transferred patients at age 21 or older (75%) [40].

This study is not without any limitations. The cross-sectional design provides a one-time assessment. Similarly, we conducted the study on an exclusive sample of young people diagnosed with chronic rheumatological conditions. Ideally, a longitudinal study on a wide spectrum of young people would have been more appropriate. Additionally, the UNC TR_x_ANSITION tools has been recommended for ages 12 years and older. However, in our study, this tool was used in children as young as 10 years, since the transition occurs earlier and the discussion of transition starts that early in our centers. Moreover, we were not able to accurately analyze demographic factors such as gender differences, household income or literacy level in transitional readiness accurately due to limited sample size. Previous studies have demonstrated that females tend to demonstrate higher transition readiness as they tend to mature earlier [21]. Other studies demonstrated positive associations for transition readiness in higher median household income [25], while parent education and household size has not shown to have significant association with transition readiness [24]. On the other hand, some of the advantages of this study is the use of a provider administered questionnaire, as opposed to a self-administered questionnaire. Hence, it does not solely rely on patient self-report, instead the validity of the patient’s responses can be assessed by the health provider either during the interview and/or by examining specific information contained in a patient’s medical chart. Also, the assessment tool uses a feedback approach to help guide health-care providers to praise the adolescent on knowledge/skills they have already mastered, help them improve on tasks they perform adequately, and help them focus on the areas where they have the lowest competencies [16].

## Conclusion

At present, AYA with chronic rheumatic diseases in Oman are not adequately prepared for the ‘transfer’ to adult care at the current cut-off age of 13 years. In addition, the transitional readiness of the older age group (> 18 years), is not considered optimal. Hence, there is a dire need to prioritize adolescent health care practice, health care facilities, clinical education and research in this region of the world. This could be accomplished through the development of adolescent health care centers that bring together expert interdisciplinary care, excellent health provider training, and cutting-edge adolescent health research to provide leadership throughout the region and further both the health of adolescents and their access to high-quality, holistic health services .

## Data Availability

The datasets used and/or analyzed during the current study are available from the corresponding author upon request.

## Abbreviations

HCT: Health Care Transition
AYA: Adolescents and Young Adults
SLE: Systemic lupus erythematosus
JIA: Juvenile Idiopathic Arthritis
GCC: Gulf Cooperation Council
AAP: American Academy of Pediatrics
AAFP: American Academy of Family Physicians
ACP: American College of Physicians
CARRA: Childhood Arthritis and Rheumatology Research Alliance
